# Correlation of liver enhancement in gadoxetic acid–enhanced MRI with liver functions: a multicenter-multivendor analysis of hepatocellular carcinoma patients from SORAMIC trial

**DOI:** 10.1007/s00330-021-08218-9

**Published:** 2021-08-31

**Authors:** Osman Öcal, Bora Peynircioglu, Christian Loewe, Otto van Delden, Vincent Vandecaveye, Bernhard Gebauer, Christoph J. Zech, Christian Sengel, Irene Bargellini, Roberto Iezzi, Alberto Benito, Kerstin Schütte, Antonio Gasbarrini, Ricarda Seidensticker, Moritz Wildgruber, Maciej Pech, Peter Malfertheiner, Jens Ricke, Max Seidensticker

**Affiliations:** 1 Department of Radiology, University Hospital, Ludwig Maximilian University of Munich, Marchioninistrasse 15, 81377 Munich, Germany; 2 Department of Radiology, Hacettepe University, Ankara, Turkey; 3 Section of Cardiovascular and Interventional Radiology, Department of Bioimaging and Image-Guided Therapy, Medical University of Vienna, Vienna, Austria; 4 Department of Radiology and Nuclear Medicine, Academic Medical Center, University of Amsterdam, Amsterdam, The Netherlands; 5 Department of Radiology, University Hospitals Leuven, Leuven, Belgium; 6 Department of Radiology, Charité – University Medicine Berlin, Berlin, Germany; 7 Radiology and Nuclear Medicine, University Hospital Basel, University of Basel, Basel, Switzerland; 8 Radiology Department, Grenoble University Hospital, La Tronche, France; 9 Department of Vascular and Interventional Radiology, University Hospital of Pisa, Pisa, Italy; 10 Dipartimento di Diagnostica per Immagini, Radioterapia Oncologica ed Ematologia, Fondazione Policlinico Universitario A. Gemelli IRCCS, UOC di Radiologia, Rome, Italy; 11 Abdominal Radiology Unit, Deparment of Radiology, Clínica Universidad de Navarra, Pamplona, Spain; 12 Department of Internal Medicine and Gastroenterology, Niels-Stensen-Kliniken Marienhospital, Osnabrück, Germany; 13 Fondazione Policlinico Gemelli IRCCS, Università’ Cattolica del Sacro Cuore, Rome, Italy; 14 Departments of Radiology and Nuclear Medicine, University of Magdeburg, Magdeburg, Germany; 15 Department of Medicine II, University Hospital, LMU Munich, Munich, Germany

**Keywords:** Magnetic resonance imaging, Gadoxetic acid, Liver function, Liver-to-spleen ratio, Hepatocellular carcinoma

## Abstract

**Objectives:**

To evaluate the correlation between liver enhancement on hepatobiliary phase and liver function parameters in a multicenter, multivendor study.

**Methods:**

A total of 359 patients who underwent gadoxetic acid–enhanced MRI using a standardized protocol with various scanners within a prospective multicenter phase II trial (SORAMIC) were evaluated. The correlation between liver enhancement on hepatobiliary phase normalized to the spleen (liver-to-spleen ratio, LSR) and biochemical laboratory parameters, clinical findings related to liver functions, liver function grading systems (Child-Pugh and Albumin-Bilirubin [ALBI]), and scanner characteristics were analyzed using uni- and multivariate analyses.

**Results:**

There was a significant positive correlation between LSR and albumin (rho = 0.193; *p* < 0.001), platelet counts (rho = 0.148; *p* = 0.004), and sodium (rho = 0.161; *p* = 0.002); and a negative correlation between LSR and total bilirubin (rho = −0.215; *p* < 0.001) and AST (rho = −0.191; *p* < 0.001). Multivariate analysis confirmed independent significance for each of albumin (*p* = 0.022), total bilirubin (*p* = 0.045), AST (*p* = 0.031), platelet counts (*p* = 0.012), and sodium (*p* = 0.006). The presence of ascites (1.47 vs. 1.69, *p* < 0.001) and varices (1.55 vs. 1.69, *p* = 0.006) was related to significantly lower LSR. Similarly, patients with ALBI grade 1 had significantly higher LSR than patients with grade 2 (1.74 ± 0.447 vs. 1.56 ± 0.408, *p < *0.001); and Child-Pugh A patients had a significantly higher LSR than Child-Pugh B (1.67 ± 0.44 vs. 1.49 ± 0.33, *p* = 0.021). Also, LSR was negatively correlated with MELD-Na scores (rho = −0.137; *p* = 0.013). However, one scanner brand was significantly associated with lower LSR (*p* < 0.001).

**Conclusions:**

The liver enhancement on the hepatobiliary phase of gadoxetic acid–enhanced MRI is correlated with biomarkers of liver functions in a multicenter cohort. However, this correlation shows variations between scanner brands.

**Key Points:**

*• The correlation between liver enhancement on the hepatobiliary phase of gadoxetic acid–enhanced MRI and liver function is consistent in a multicenter-multivendor cohort.*

*• Signal intensity–based indices (liver-to-spleen ratio) can be used as an imaging biomarker of liver function.*

*• However, absolute values might change between vendors.*

**Supplementary Information:**

The online version contains supplementary material available at 10.1007/s00330-021-08218-9.

## Introduction

Gadoxetic acid is a hepatocellular specific contrast agent showing selective hepatocyte uptake, which peaks approximately 20 min after injection (hepatobiliary phase) [1]. Hepatobiliary phase images increase the detection rate of primary and secondary liver tumors by enhancing contrast differences between lesions and healthy liver tissue [2, 3]. Gadoxetic acid–enhanced MRI has been shown to improve treatment decisions based on the tumor extent compared to CT [4]. Furthermore, the degree of hepatic enhancement has been shown to be lower in patients with poor liver function. Previous studies have shown that hepatic enhancement is correlated with ALBI-score [5, 6], Child-Pugh grade [7], MELD-score [8], hypoalbuminemia [8–10], hyperbilirubinemia [9–11], INR [9], platelet count [9], serum sodium [8], and ascites [8]. However, all of these comprised single-center cohorts with a small number of patients. Additionally, the signal intensity of the liver is influenced by a variety of factors, including scanner type, field strength, and imaging sequence parameters [12]. Okada et al reported the first multicenter study on the correlation between biochemical parameters and hepatobiliary phase enhancement using the liver-to-spleen ratio (LSR) and showed that prothrombin activity, bilirubin, and total cholesterol levels are significantly associated with the liver enhancement in an Asian cohort [13]. There was no difference in signal intensity between 1.5- and 3.0-T scanners. Nonetheless, all patients within this study underwent MRI with machines from the same vendor. Recently, a single-center study showed that signal intensity (SI)–based indices (including LSR) remained similar when the same patient underwent consecutive MRI within the same center and identified no significant difference between scanners from the same vendor [14]. However, evaluation of the consistency of SI-based indices between different scanner brands lacks in the literature.

The objective of this post hoc study was to evaluate if the correlation between liver enhancement on gadoxetic acid MRI and liver function is preserved in a multivendor study cohort comprised of hepatocellular carcinoma (HCC) patients collected within a prospective randomized controlled trial (SORAMIC, SORAfenib in combination with local MICro-therapy guided by gadolinium-EOB-DTPA-enhanced MRI; EudraCT 2009-012576-27, NCT01126645) performed in multiple centers from various countries.

## Material and methods

### Study population

SORAMIC was a multicenter randomized-controlled phase II trial conducted at 38 sites in 12 countries. Within the palliative arm of the study, 424 patients with intermediate or advanced stage HCC (BCLC-B and C) were randomized either to sorafenib monotherapy or Y-90 radioembolization followed by sorafenib treatment between January 2011 and February 2016. Inclusion and exclusion criteria and results of the study were previously published [15]. In terms of liver functions, patients with Child-Pugh scores A to B7 were allowed to be included in the SORAMIC trial. For inclusion into this substudy, the presence of MRI with gadoxetic acid before randomization was necessary. The study protocol was approved by the institutional review boards of each participating center, and written informed consent was acquired from each patient, including a collection of baseline images for centralized analysis.

Of the 424 patients randomized within the SORAMIC trial, 372 patients had baseline MRI images, including the hepatobiliary phase, available. Four patients were excluded due to a history of splenectomy, eight patients due to missing baseline albumin or bilirubin values, and one patient due to significant artifacts precluding SI measurement in the spleen. The study population consisted of the remaining 359 patients from 31 centers out of eleven countries.

### Imaging protocol

Patients underwent MRI with a standardized imaging protocol according to the diagnostic study of the SORAMIC trial [4]. Brands, models, field strengths of the scanners used in participating centers, and imaging parameters from exemplary centers recruited the highest number of patients for each scanner brand are listed in Supplementary tables 1 and 2. The MRI protocol consisted of pre-contrast T1-weighted gradient echo (GRE) sequences acquired 2D and 3D which was followed by an injection of 0.1 ml/kg gadoxetic acid and the dynamic series. At 20 min after contrast injection, T1-weighted GRE 2D and 3D hepatobiliary phase images were acquired. During the interval between dynamic series and hepatobiliary phase, T2-weighted turbo spin-echo 2D sequences and diffusion-weighted imaging (not mandatory) were performed.

### Image analysis

Image analysis was performed by a radiologist with 6 years of experience in abdominal imaging who was blinded to all clinical parameters. SI of the liver and spleen on T1-weighted GRE 3D hepatobiliary phase images were calculated using a circular or oval ROI with a size of approximately 250 mm^2^. Large vessels, bile ducts, tumor lesions, and major artifacts were avoided during ROI placement. In order to sample the whole liver, two of the left liver lobe, right posterior sector, and right anterior sector were chosen (to exclude segments replaced with tumors). Then, the mean SI of the liver was measured separately three times in each of two, and the average SI was recorded for each measurement. LSR was calculated with the following formula:

$$ \mathrm{LSR}=\frac{{\mathrm{SI}}_{\mathrm{post}}\ \mathrm{of}\ \mathrm{the}\ \mathrm{liver}}{{\mathrm{SI}}_{\mathrm{post}}\ \mathrm{of}\ \mathrm{the}\ \mathrm{spleen}\ } $$

Additionally, the presence of ascites, pleural effusion, and gastroesophageal varices in the images have been recorded (as present, not present).

### Clinical laboratory parameters

All patients underwent clinical examination, complete blood count, and serum biochemical laboratory assessments at the participating centers before randomization. The diagnosis of cirrhosis was made by the local investigator based on the history, imaging, and clinical findings. The following parameters were included in this analysis: age, sex, body mass index (BMI), hemoglobin, white blood cell count, thrombocyte count, total bilirubin, albumin, alanine transaminase (ALT), aspartate transaminase (AST), gamma-glutamyl transpeptidase (GGT), alkaline phosphatase (ALP), serum creatinine, and the international normalized ratio (INR). The Child-Pugh grade of the patients was also reported by centers. Additionally, MELD-Na and ALBI scores were calculated for each patient using the following formulas:

$$ {\displaystyle \begin{array}{c}\mathrm{MELD}=\left(0.957\ast \mathit{\ln}\left(\mathrm{creatinine}\right)\right)+\left(0.378\ast \mathit{\ln}\left(\mathrm{bilirubin}\right)\right)+\Big(\left(1.12\ast \mathit{\ln}(INR)\right)+0.643\\ {}\mathrm{MELD}- Na=\mathrm{MELD}-\mathrm{serum} Na-\left(0.025\ast \mathrm{MELD}\ast \left(140-\mathrm{sodium}\right)\right)+140\\ {}\mathrm{ALBI}\ \mathrm{score}=\left({\mathit{\log}}_{10}\mathrm{bilirubin}\ast 0.66\right)+\left(\mathrm{albumin}\ast -0.085\right)\end{array}}, $$

and MELD-Na score was rounded to nearest integer, and patients with an ALBI score of ≤ −2.60 were graded as 1, > −2.60 and ≤ −1.39 as 2, and > −1.39 as 3.

### Statistical analysis

Analyses were performed using R statistical software (R version 3.6.3). Categorical variables were reported as counts and percentages, and continuous variables as means and standard deviations or medians and interquartile ranges. Univariate analyses for correlation of LSR with categorical variables were carried out using unpaired t-tests and one-way analysis of variance (ANOVA) tests. Non-parametric univariate correlation analysis (Spearman’s rank-order correlation) was performed for LSR and the clinical parameters. A *p* value < 0.05 was regarded as statistically significant. Variables with significant correlation were included in multivariate analysis. Patients with missing data were excluded from the multivariate analysis. We used the receiver operating characteristic (ROC) curve to determine the cut-off values for different scanners that could produce the highest sensitivity and specificity to predict higher ALBI grade.

## Results

Baseline characteristics of the patients are summarized in Table 1. Out of 359 patients, 8 (2.2%) had initial (BCLC-A), 108 (30.1%) had intermediate (BCLC-B), and 243 (67.7%) had advanced (BCLC-C) HCC. Macrovascular invasion was present in 182 (50.6%) patients. A total of 286 (79.6%) patients had liver cirrhosis, 327 (91.1%) patients had Child-Pugh A liver functions, and 32 (8.9%) patients had B. Underlying liver disease was hepatitis B in 28 (10.6%), hepatitis C in 75 (24.4%), alcoholic liver disease in 125 (40.2%), hepatitis B and C in 1 (0.2%), hepatitis B and alcoholic liver disease in 8 (2.2%), and hepatitis C and alcoholic liver disease in 12 (3.3%), non-alcoholic fatty liver disease in 43 (11.9%), cryptogenic in 42 (11.6%), and hemochromatosis in 11 (3.0%) patients. Results of baseline laboratory values are summarized in Table 2. In total, 339 (94.4%) patients were imaged with a 1.5-T scanner and 20 (5.6%) patients with a 3-T.

**Table 1:** Baseline patient characteristics

	***n* = 359**
**Age (median, range)**	67, 41-84
**Gender (male)**	312 (86.9%)
**Body mass index (median, range)**	26.5 (16.0-42.2)
**Cirrhosis (yes)**	286 (79.6%)
**Underlying liver disease**	
Hepatitis B	28 (10.6%)
Hepatitis C	75 (24.4%)
Alcoholic liver disease	125 (40.2%)
Hepatitis B and hepatitis C	1 (0.2%)
Hepatitis B and alcoholic liver disease	8 (2.2%)
Hepatitis C and alcoholic liver disease	12 (3.3%)
NAFLD	43 (11.9%)
Cryptogenic	42 (11.6%)
Hemochromatosis	11 (3.0%)
**Child-Pugh grade**	
A	327 (91.1%)
B	32 (8.9%)
**Macrovascular invasion (yes)**	182 (50.6%)
**Ascites**	61 (16.9%)
**Pleural effusion**	11 (3.0%)
**Varices**	98 (27.2%)
**BCLC**	
A	8 (2.3%)
B	108 (30.0%)
C	243 (67.7%)
**MRI scanner brand**	
GE	64 (17.8%)
Philips	137 (38.2%)
Siemens	149 (41.5%)
Toshiba	9 (2.5%)
**MRI magnetic field strength**	
1.5	339 (94.4%)
3	20 (5.6%)
**Countries (number of centers)**	
Austria (2)	13
France (6)	68
Germany (7)	131
Italy (3)	27
Netherlands (1)	32
Poland (3)	30
Slovenia (1)	12
Spain (1)	13
Switzerland	3
United Kingdom (5)	26
Turkey (1)	4

**Table 2:** Summary of baseline parameters

Parameter	No. of patients	Median	Range	1^st^ Quartile	3^rd^ Quartile
LSR	359	1.59	0.66 – 4.25	1.35	1.86
Albumin (g/L)	359	39.0	24.0 – 60.0	35.85	42.0
Bilirubin (μmol/L)	359	14.1	2.0 – 51.0	9.8	19.9
INR	321	1.1	0.63 – 4.34	1.0	1.2
ALT (ukat/L)	356	0.78	0.11 – 5.21	0.48	1.22
AST (ukat/L)	344	1.01	0.23 – 9.6	0.73	1.58
GGT (ukat/L)	355	3.55	0.16 – 41.1	1.85	6.7
ALP (ukat/L)	353	2.38	0.43 – 18.6	1.6	3.71
Platelet count (×10^9^/L)	359	168	25 – 787	122.5	219.5
Hemoglobin (g/dL)	359	8.56	5.7 – 16.5	7.6	9.2
Leukocytes (×10^9^/L)	359	6.4	0.52 – 17.5	5.0	7.97
Total protein (g/L)	345	75.7	60.6 – 98.0	71.0	80.0
Creatinine (μmol/L)	356	74.2	43.0 – 176.8	64.0	88.4
BUN (mmol/L)	310	5.35	0.74 – 22.8	4.1	7.45
MELD-Na score	321	8	6-23	7	9
ALBI score	359	−2.57	−4.13 to −1.30	−2.90	−2.19

*ALBI*, albumin-bilirubin; *ALP*, alkaline phosphatase; *ALT*, alanine transaminase; *AST*, aspartate transaminase; *BMI*, body mass index; *GGT*, gamma-glutamyl transpeptidase; *LSR*, liver-to-spleen ratio; *INR*, international normalized ratio

### Correlation of LSR with patient and tumor characteristics

Univariate analysis revealed no significant correlation between LSR and age (*p* = 0.186), body mass index (*p* = 0.288), and gender (*p* = 0.579).

Similarly, there was no significant correlation between LSR and the presence of macrovascular invasion (1.66 ± 0.48 vs. 1.65 ± 0.37, *p* = 0.853), BCLC stage (1.74 for A, 1.66 for B, and 1.65 for C; *p* = 0.825), and the ECOG performance score of patients (0 vs. ≥ 1; *p* = 0.773).

### Correlation of LSR with laboratory parameters

**Fig. 1:**
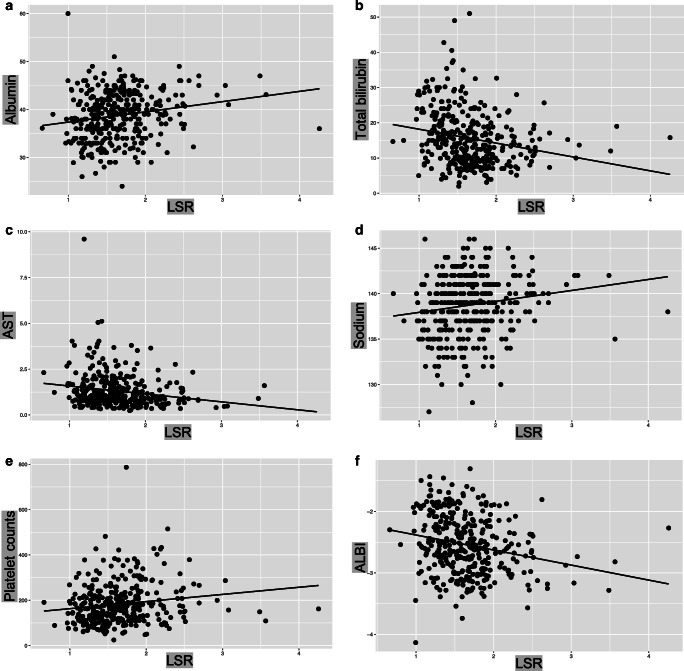
Correlation between LSR and biochemical parameters. Scatterplots with linear regression lines showing LSR versus with the variables with statistical significance: **a** Albumin (rho = 0.193, *p* < 0.001), (**b**) total bilirubin (rho = −0.215, *p* < 0.001), (**c**) AST (rho = −0.191, *p* < 0.001), (**d**) sodium (rho = 0.161, *p* = 0.002), (**e**) platelet count (rho = 0.148, *p* = 0.004), and (**f**) ALBI score (rho = −0.225, *p* < 0.001). ALBI, albumin-bilirubin; AST, aspartate transaminase; LSR, liver-to-spleen ratio

Results of the correlation analysis between LSR and laboratory parameters are listed in Table 3. Univariate analysis revealed a positive correlation between LSR and albumin (rho = 0.193; *p* < 0.001; Figure 1a), platelet counts (rho = 0.148; *p* = 0.004; Figure 1b), and sodium (rho = 0.161; *p* = 0.002; Figure 1c). In addition to this, there was a significant negative correlation between LSR and total bilirubin (rho = −0.215; *p* < 0.001; Figure 1d) and AST (rho = −0.191; *p* < 0.001; Figure 1e).

**Table 3:** Univariate and multivariate analysis of the correlation between LSR and clinical laboratory parameters

Parameter	Univariate analysis		Multivariate analysis (*n* = 335)		
	Spearman’s rho	*p* value	Estimate	Standard error	*p* value
Albumin	0.193	**< 0.001**	0.010	0.004	**0.022**
Bilirubin	−0.215	**< 0.001**	−0.006	0.003	**0.045**
INR	−0.030	0.584			
ALT	−0.063	0.228			
AST	−0.191	**< 0.001**	−0.053	0.024	**0.031**
GGT	−0.038	0.472			
ALP	−0.085	0.108			
Total protein	−0.056	0.297			
Platelet count	0.148	**0.004**	0.0007	0.0002	**0.012**
Hemoglobin	0.096	0.067			
Leukocytes	0.050	0.341			
Creatinine	−0.014	0.786			
Sodium	0.161	**0.002**	0.018	0.006	**0.006**
BUN	−0.036	0.520			
Patient age	0.069	0.186			
Body mass index	−0.057	0.292			
MELD score*	−0.137	**0.013**	−	-	-
ALBI score**	−0.225	**< 0.001**	−	**-**	**-**

*ALBI*, albumin-bilirubin; *ALP*, alkaline phosphatase; *ALT*, alanine transaminase; *AST*, aspartate transaminase; *BMI*, body mass index; *GGT*, gamma-glutamyl transpeptidase; *INR*, international normalized ratio

*MELD score was not included in the multivariate analysis due to interference with bilirubin

**ALBI score was not included in the multivariate analysis due to interference with albumin and bilirubin

Multiple regression analysis with 335 patients confirmed the significant correlation between LSR and albumin (*p* = 0.033), total bilirubin (*p* = 0.034), AST (*p* = 0.024), platelet counts (*p* = 0.009), and sodium (*p* = 0.004).

### Correlation of LSR with clinical features related to liver functions

The presence of cirrhosis was significantly correlated with lower LSR (1.62 ± 0.43 vs. 1.77 ± 0.4, *p* = 0.009). Ascites was present in 61 (16.9%), pleural effusion in 11 (3.0%), and varices in 98 (27.2%) patients. Patients with ascites had significantly lower LSR than patients without (1.69 vs. 1.47, *p* < 0.001), as well as varices (1.69 vs. 1.55, *p* = 0.006). Although the patients with pleural effusion had lower LSR, the difference was marginally non-significant (1.66 vs. 1.39, *p* = 0.056).

### Correlation of LSR with liver function scoring systems

There was a significant negative correlation between the LSR and ALBI scores (rho = −0.225; *p* < 0.001; Figure 1f), and patients with ALBI grade 1 had significantly higher LSR than patients with grade 2 (1.74 ± 0.447 vs. 1.56 ± 0.408, *p* < 0.001). Similarly, there was a significant negative correlation between LSR and MELD-Na scores (rho = −0.137; *p* = 0.013). Patients with Child-Pugh score A had significantly higher LSR than patients with Child-Pugh B (1.67 ± 0.44 vs. 1.49 ± 0.33, *p* = 0.021).

### Correlation of LSR with scanner characteristics

There was no significant difference in LSR between patients who underwent MRI with 1.5-T and 3-T scanners (1.65 ± 0.43 vs. 1.74 ± 0.45, *p = *0.853).

**Fig. 2:**
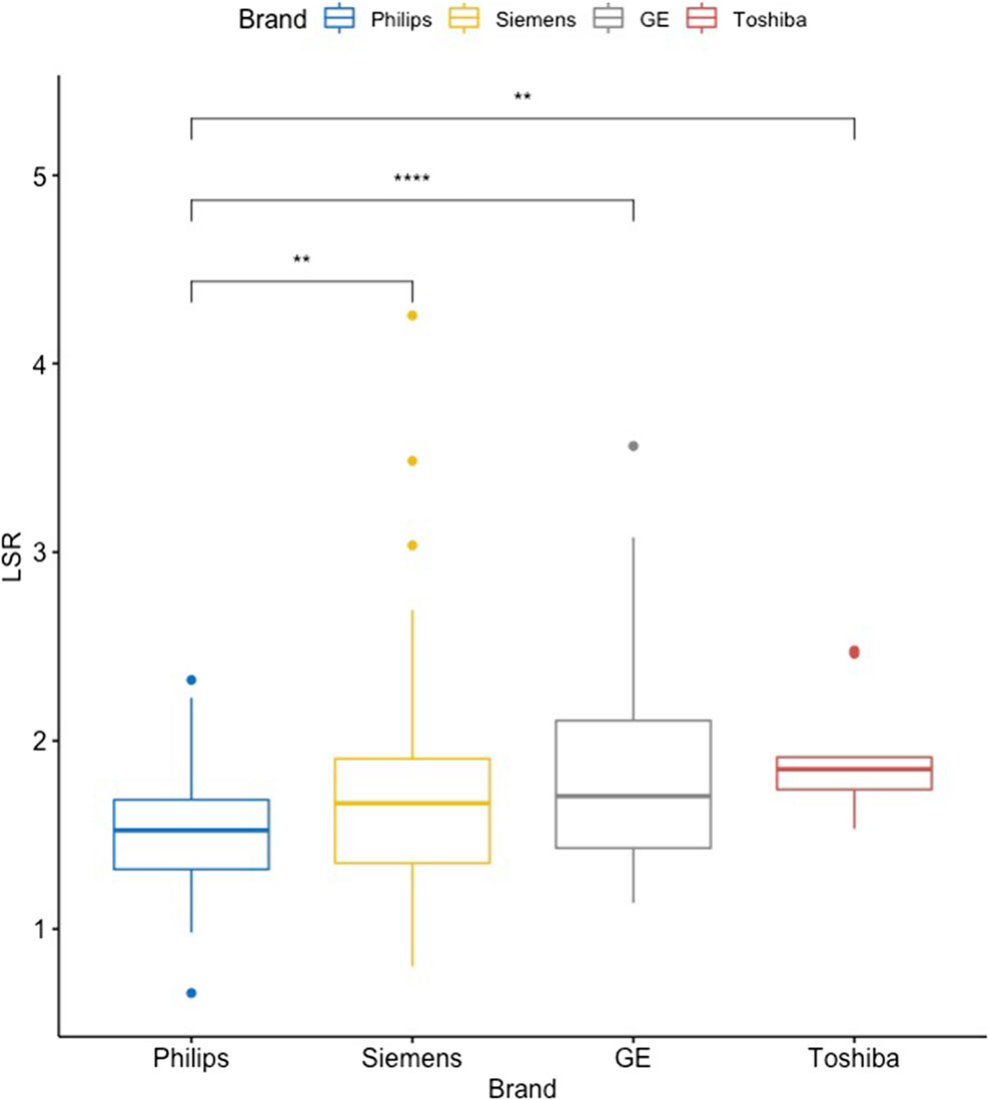
Comparison of LSR between scanner brands. Asterisks show *p* values for comparison of LSR between each brand and Philips. ** 0.001−0.01, ****< 0.0001

However, patients scanned with one scanner brand (Philips) had significantly lower LSR than all the other three brands (*p* < 0.001; Figure 2). In order to eliminate possible differences in liver functions between scanner brands, a separate multivariate model was created including scanner brand in addition to parameters having a statistically significant correlation with LSR. This model confirmed that scanner brand is independently associated with LSR, and other parameters preserved significant correlation with LSR as in the initial model (Supplementary table 3). There was no significant difference between the other brands. By ROC curve analysis revealed a cut-off value of 1.46 for LSR to have the highest sensitivity (55.2%) and specificity (67.1%) to predict ALBI grade ≥ 2 for Philips scanners, and 1.66 with a sensitivity of 57.9% and specificity of 63.1% for other scanners (Supplementary figure 1).

## Discussion

Our results show that LSR, a hepatobiliary phase MRI SI-based parameter, maintains the correlation with biochemical parameters of liver function and liver function grading systems in a large cohort collected within a multicenter, multivendor study. This finding supports the routine clinical usage of hepatobiliary phase images as an imaging marker of liver functions. In addition to laboratory parameters, liver enhancement on the hepatobiliary phase could be used as a surveillance parameter of liver parenchymal functional status in patients with cirrhosis. Additionally, it could serve in the complex decision-making process of HCC treatment, especially as a decision aid in favor of resection or local ablation in the early stage of HCC disease by predicting post-hepatectomy liver failure with estimation of the functional reserve capacity. However, although this correlation is preserved between scanners, absolute LSR values differ between vendors. One brand had significantly lower LSR values than other brands, and this difference was preserved in a multivariable analysis considering other biochemical parameters correlated with LSR.

There was a significant correlation between LSR and several clinical laboratory parameters in our study, including albumin, total bilirubin, AST, platelet count, and sodium. LSR was also correlated with imaging findings related to deteriorated liver functions, such as ascites and varices. All of these parameters are known to correlate with liver function and incorporated in survival prediction systems in patients with chronic liver diseases [16–21]. Furthermore, LSR had a significant correlation with the two most commonly used liver function grading systems [18, 19, 22].

Gadoxetic acid has an intra- and extravascular compartment distribution in arterial and portal venous phases, similar to other gadolinium-based contrast media; however, it is actively taken up by hepatocytes via organic anion transporting polypeptides (OATP1B1/3) during the transitional and hepatobiliary phases [23]. Increased contrast between lesions and parenchyma in the hepatobiliary phase has improved treatment decisions in primary and secondary liver tumors [2, 4, 24, 25]. Liver enhancement on the hepatobiliary phase depends on the concentration of OATPs in the liver, which has been shown to decrease as fibrosis progresses, as well as the total number of hepatocytes [26]. Additionally, Na^+^ - taurocholate cotransporting polypeptide (NTCP), which influxes two sodium ions and one conjugated bile salt into hepatocytes, is shown to uptake gadoxetic acid with higher affinity but lower capacity than OATPs [27]. By these features, gadoxetic acid–enhanced MRI offers the possibility to evaluate liver functions with additional advantages over biochemical markers or other imaging-based assessments, including the possibility to assess regional liver functions [28]. However, SI measurements are relative parameters that depend on many technical parameters; therefore, these values cannot be used to compare consequent studies of the patient or different patients. Several SI-based parameters have been described to overcome these variations by correcting liver enhancement with spleen or muscle intensities. Previous studies have shown a good to a perfect positive correlation between LSR, relative liver enhancement, contrast uptake index, and hepatocyte uptake index in patients with chronic liver disease (0.715–0.906), as well as a good to a perfect intra- and interobserver correlation for each parameter [6].

Several studies have shown that these parameters are significantly correlated with biochemical markers of liver function, the presence of liver disease, the degree of liver fibrosis, and liver function scoring systems [5, 7–9]. However, most of these studies were single-center retrospective cohorts with a limited number of patients. A multicenter study performed in Japan using the same scanner brand showed that sufficient liver enhancement (LSR > 1.5) is correlated with prothrombin activity, total bilirubin, and total cholesterol levels [13]. More recently, a single-center study showed that liver enhancement parameters (LSR, relative enhancement, liver-to-muscle ratio) are consistent between consecutive imaging with no difference between scanners using different field strengths from the same brand [14]. Our study confirmed the correlation between signal intensity-based assessments (LSR) and liver function in a multicenter, multivendor study using a much broader variability in scanner brands and field strengths. However, probably due to variations and heterogeneities between centers, scanner brands, and different etiologies of underlying liver diseases, the correlation was weaker than previously published results. For example, the correlation coefficient of LSR with ALBI score was −0.225 in our study, while Takatsu et al and Beer et al reported −0.61 and −0.491 [5, 6].

Additionally, our study showed that despite the significant correlation between LSR and liver function tests, absolute LSR values differ between vendors. Patients who underwent MRI with one vendor showed significantly lower LSR than other brands, and this difference was confirmed in a separate multivariate analysis considering liver function. We evaluated imaging parameters for scanner brands, and despite slight variations, we believe the difference in absolute LSR values results from different post-processing software. However, it did not affect the correlation with liver function parameters as described above. This indicates that the scanner brand should be taken into account when absolute levels are used in decision making, such as sufficient liver enhancement (LSR > 1.5). Additionally, explorative ROC analysis to define cut-off values for LSR discriminating ALBI grade identified 1.46 for Philips and 1.66 for other brands to have the highest sensitivity and specificity. Another interesting finding in our study was the significant correlation between LSR and serum sodium levels. Although hyponatremia is common at advanced stages of cirrhosis and incorporated into the MELD-Na score, a decrease in NCTP activity in patients with low serum sodium might also be contributing and requires further analysis.

HCC was the fourth common cause of cancer-related death worldwide in 2018 [29], and its incidence is expected to increase, especially in Western countries [30]. In the Western population, up to 90% of the cases develop within the background of chronic liver disease, and besides tumor extent, the liver function of the patient is the primary determinant of outcome [31]. Several staging systems have been developed to predict outcomes and plan optimal treatment, and all have incorporated various parameters related to the liver function of the patient [32–34]. Gadoxetic acid MRI provides better treatment allocation than CT considering tumor burden [4], and, in addition to this, information related to liver function. It has been shown that liver function assessment by gadoxetic acid–enhanced MRI can predict post-hepatectomy liver failure [35, 36], can be used to plan hepatectomy by evaluation of regional liver function [37], and is superior to the indocyanine clearance test in predicting complications [38]. Additionally, some novel imaging parameters related to tumor biology (i.e., presence of peritumoral hypointensity) can be evaluated on hepatobiliary phase images [39]. As a result of these advantages, gadoxetic acid–enhanced MRI has been the primary evaluation tool in HCC patients [40]. Our research adds further evidence to the implementation of gadoxetic acid–derived liver function assessment in patients with hepatocellular carcinoma [41].

Our study has several limitations. First, despite the standardized MRI imaging protocol within the prospective imaging study, there were variations in imaging parameters (TE, FA, fat-saturation techniques) at centers. However, all centers obtained the 3D T1-weighted fat-sat sequences with a fixed time delay for the hepatobiliary phase. Further on, liver function assessment was based on a relative parameter using the ratio of liver and spleen measurements obtained at the same phase and same slice, which should be robust against these variabilities. Second, ROI placement may have caused variations related to the heterogeneity of liver functions in different segments. To overcome this limitation, multiple ROIs were placed at different liver segments to increase sampling volume, and there was a perfect correlation (rho = 0.975) between two measurements obtained within the liver (not shown). Third, all patients had relatively preserved liver function with no patients having Child-Pugh class C, which narrows the range of LSR in our cohort and limits to evaluate changes in liver enhancement in patients with worse liver functions. Also, there was only one reader, and only one of the signal intensity-based indices (LSR) was used. However, LSR was one of the most commonly used parameters and is robust to calculate, with measurements from liver and spleen done within the same slice (unlike liver-to-muscle ratio), and as pointed out above, previous studies have shown perfect intra- and interobserver correlation of LSR [6]. Finally, the number of patients imaged with 3.0T scanners was low (*n* = 20), and we did not find any difference in LSR between patients scanned with different field strengths. Although the data is not sufficiently powered to make a statement on the field strength, this result was in agreement with previous reports [13]. Additionally, this analysis aimed to confirm if the correlation is preserved in a multivendor cohort instead of comparing the different parameters of a MRI-based liver function assessment.

In conclusion, the correlation of liver enhancement on the hepatobiliary phase of gadoxetic acid–enhanced MRI with liver function is maintained between several centers and field strengths in patients with HCC, with or without underlying liver cirrhosis. This underlines the importance of gadoxetic acid–enhanced MRI in the treatment decision-making process as an imaging marker of liver functions.

## Supplementary information

ESM 1 (DOCX 225 kb)

## Abbreviations

ALBI: Albumin-bilirubin
ALP: Alkaline phosphatase
ALT: Alanine transaminase
AST: Aspartate transaminase
BCLC: Barcelona clinic liver cancer
BMI: Body-mass index
GGT: Gamma-glutamyl transpeptidase
GRE: Gradient echo
INR: International normalized ratio
LSR: Liver-to-spleen ratio
MELD: Model of end-stage liver disease
OATP: Organic anion transporting polypeptides

## Declarations

### Guarantor

The scientific guarantor of this publication is Max Seidensticker.

### Statistics and biometry

One of the authors (OÖ) has significant statistical expertise.

### Study subjects or cohorts overlap:

The main results of the clinical trial have been previously reported in *Ricke J Hepatology 2019.*

### Methodology

• retrospective

• diagnostic or prognostic study

• multicenter study
