# Supplementary material for: Correlation of liver enhancement in gadoxetic acid–enhanced MRI with liver functions: a multicenter-multivendor analysis of hepatocellular carcinoma patients from SORAMIC trial
Source: Eur Radiol. 2021 Aug 31;32(2):1320–9. doi: 10.1007/s00330-021-08218-9 (PMC8795026; doi:10.1007/s00330-021-08218-9)

**Supplementary table 1. Scanner brands and models used in centers**

| **Brand** | **Scanner** | **Field strength** | **No. of patients** |
| --- | --- | --- | --- |
| GE | Discovery  Discovery  Signa | 1.5  3.0  1.5 | 6  1  57 |
| Philips | Achieva  Ingenia  Intera | 1.5  3.0  1.5 | 54  2  81 |
| Siemens | Aera  Avanto  Skyra  Symphony  Tim Trio | 1.5  1.5  3.0  1.5  3.0 | 53  65  7  14  10 |
| Toshiba | Mrt200sp5 | 1.5 | 9 |

**Supplementary table 2. Imaging parameters in different scanners from exemplary centers**

|  | **Philips-1**  **N=73** | **Philips-2**  **N=28** | **Siemens-1**  **N=32** | **Siemens-2**  **N=18** | **GE-1**  **N=16** | **GE-2**  **N=12** | **Toshiba**  **N=9** |
| --- | --- | --- | --- | --- | --- | --- | --- |
| **TR** | 4.03 | 4.03 | 5.77 | 4.36 | 5.2 | 3.77 | 4.8 |
| **TE** | 1.95 | 1.95 | 2.55 | 1.92 | 2.5 | 1.7 | 1.9 |
| **Flip angle** | 10 | 10 | 10 | 9.9 | 12 | 13 | 15 |
| **Slice thickness** | 6 | 6 | 5 | 3 | 5 | 3.6 | 8 |
| **Slice interval** | 3 | 3 | 1.7 | 1.4 | 5 | 1.8 | 4 |
| **Matrix** | 180x178 | 172x174 | 224x126 | 320x165 | 320x192 | 256x192 | 256x168 |
| **Receiver bandwidth** | 434 | 434 | 251 | 400 | 244.1 | 244.1 |  |
| **Echo train length** | 41 | 43 | 1 | 1 | 1 | 1 | 1 |

**Supplementary table 3.** **Multivariate analysis of the correlation between LSR and clinical variables including brand**

|  | **Estimate** | **Std. Error** | ***P*-value** |
| --- | --- | --- | --- |
| ALBI score | -0.1508 | 0.046 | **0.001** |
| AST | -0.0715 | 0.023 | **0.002** |
| Platelets | 0.0008 | 0.0002 | **<0.001** |
| Sodium | 0.0204 | 0.006 | **0.001** |
| Brand (Philips) | -0.2575 | 0.041 | **<0.001** |

ALBI, albumin-bilirubin; AST, aspartate transaminase.

Supplementary Figure


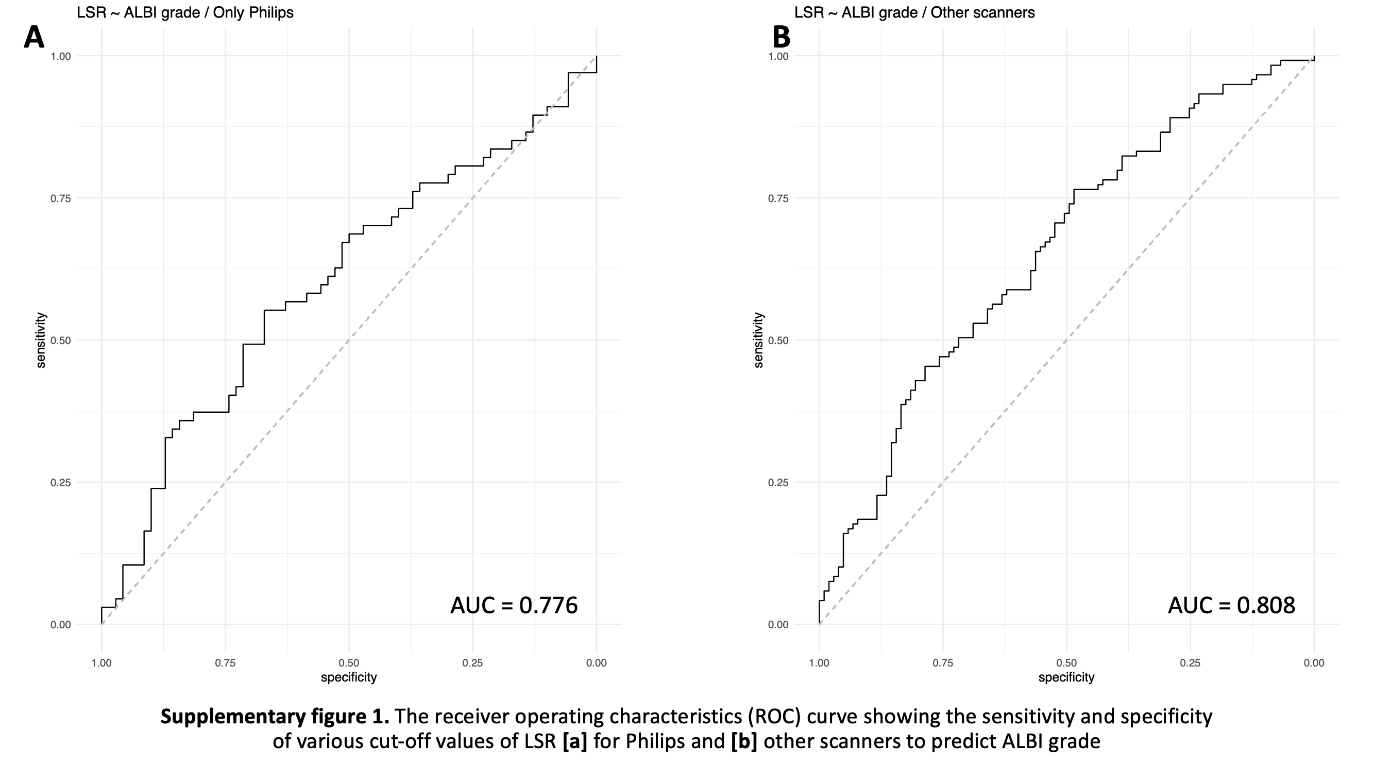

Supplement: Supplementary file 1 — (DOCX 225 kb) [file 330_2021_8218_MOESM1_ESM.docx]
